# Development and Validation of Protocol Based on Brazilian Dietary Guidelines for Adults with Diabetes Mellitus Who Attended Primary Health Care

**DOI:** 10.3390/ijerph20105784

**Published:** 2023-05-10

**Authors:** Maísa Miranda Araújo, Nathalia Pizato, Lorrany Santos Rodrigues, Laila Santos de Andrade, Verena Duarte de Moraes, Kênia Mara Baiocchi de Carvalho, Eliane Said Dutra, Patrícia Borges Botelho, Vivian Siqueira Santos Gonçalves

**Affiliations:** 1 Graduate Program in Human Nutrition, Department of Nutrition, University of Brasilia, Campus Darcy Ribeiro, Brasília 70910-900, Brazil; 2 Graduate Program in Public Health, Department of Nutrition, University of Brasilia, Campus Darcy Ribeiro, Brasília 70910-900, Brazil; 3 Nutrition and Health Research Group–PENSA, Department of Nutrition, University of Brasilia, Campus Darcy Ribeiro, Brasília 70910-900, Brazil; 4 Oswaldo Cruz Foundation, Laboratory of Regional Endemic Situations, Rio de Janeiro 21045-900, Brazil

**Keywords:** primary health care, dietary guidelines, practice guidelines, diabetes mellitus, validation study

## Abstract

Background: To date, there is no protocol providing dietary guidelines to assist health care professionals in counseling Brazilian individuals with Diabetes Mellitus (DM) assisted in primary health care (PHC) according to the Dietary Guidelines for the Brazilian Population (DGBP). Therefore, this study aimed to develop and validate a protocol based on the DGBP for health care, non-nutritionist professionals in counseling adults with DM in PHC. Methods: We systematized the recommendations published in the DGBP, the Diabetes Brazilian Society guidelines, and the scientific literature regarding food and nutrition needs of adults with DM. The clarity and relevance were validated by an expert panel (*n* = 19) and the understanding and applicability were validated by PHC professionals (*n* = 12). The degree of agreement of the experts was assessed using a Content Validity Index (CVI). Items receiving CVI > 0.8 were considered appropriate. Results: The protocol consisted of six dietary recommendations that encouraged the daily consumption of beans, vegetables, and fruits, advised the avoidance of sugar-sweetened beverages and ultra-processed foods, stimulated eating in appropriate environments, and gave additional guidance addressed to the particularities of DM. The protocol clarity, relevance, and applicability were successfully validated. Conclusion: The protocol supports health care, non-nutritionist professionals in the guidance of dietary recommendations and promoting adequate and healthy eating habits for adults with DM in PHC.

## 1. Introduction

Diabetes mellitus (DM) is a group of metabolic diseases characterized by hyperglycemia due to inadequate insulin secretion and/or action on target cells [1]. In these groups, the most frequent types of DM are type 2, a chronic disease generally associated with obesity [1]. This chronic condition represents a major impact on the quality of life and well-being of people, families, and societies worldwide. A global prevalence has been estimated of 537 million (10.5%) people aged 20–79 years with DM in 2021; in addition, DM is expected to reach 643 million people (11.3%) by 2030 and 783 million (12.2%) by 2045 [2]. In Brazil, a prevalence was estimated of 15.7 million adults (20–79 years) with DM in 2021, and it is expected to increase to 23.2 million in 2045 [2]. 

People with DM present a high risk of developing several diseases due to the long-term effects of hyperglycemia, such as retinopathy, nephropathy, neuropathy, and cardiovascular diseases [1]. Approximately 6.7 million adults are estimated to have died because of DM or its complications in 2021 [2]. To manage this disease and prevent the related complications, as well as avoid early mortality and improve the quality of life, is crucial to maintain adequate serum glucose control. Fundamental to achieving treatment goals are DM self-management education and support, and medical, nutritional, physical activity, and psychosocial care [3]. 

In this sense, Primary Health Care (PHC) plays an essential role in managing this chronic disease’s risk reduction in the global and specific Brazilian population. Brazilian PHC incorporates the Family Health Strategy (FHS), which provides multidisciplinary health assistance in addressing health promotion and disease risk reduction to population groups. FHS-trained health professionals ensure the evaluation and diagnosis of diabetes, together with individualized medical follow-up care, medication assistance, oral health care, nursing, nutrition, and physical counseling [4].

One of the instruments to support dietary counseling in the Brazilian Unified Health System (in Portuguese: Sistema Único de Saúde (SUS)) is the Dietary Guidelines for the Brazilian Population (DGBP). These Guidelines summarize the dietary recommendations on healthy eating for the Brazilian population from a holistic perspective of food (biological, cultural, social, and environmental) based on scientific evidence [5]. The DGBP was published in 2014 according to the NOVA food classification [6] and is focused on people in general. To date, there is no specific dietary guideline in the PHC according to the DGBP that is specific for adults with DM. 

Therefore, considering the crucial role of dietary intake for DM management and the need for instruments to assist health care professionals, among them non-nutritionists, in counseling Brazilian individuals with DM assisted by PHC, our study aimed to develop and validate a protocol for the use of the DGBP in dietary guidelines for adults with DM. 

## 2. Materials and Methods

The development and validation of the dietary protocol based on DGBP to adults with DM were elaborated by experiences in the food guidelines, health professionals of PHC, and DM researchers who declared themselves free of conflicts of interest, following the five steps proposed by Louzada [7]. 

### 2.1. Protocol Format Definition

Guidelines for clinical practice protocol elaboration and published Brazilian protocols were analyzed to identify the most appropriate format to guide dietary guidelines for use in individual appointments in PHC.

### 2.2. Definition of the Instrument for the Assessment of Food Consumption Markers

A bibliographic search was carried out on previous instruments for assessing food consumption in PHC to identify the best instrument to support decision-making in individual consultations.

### 2.3. Extracting Recommendations from Brazilian Dietary Guidelines

A systematic reading of the DGBP and the Brazilian Society of Diabetes guidelines [8] was carried out by two researchers, considering the target population, the context of primary health care, and the specificities of the disease, in conformity with the AGREE checklist of clinical practice guidelines [9] to identify the main recommendations that apply to people with DM. The final list of recommendations was reviewed by the research team.

### 2.4. Evidence Systematization on the Food and Nutrition Needs of Adults with DM

A literature review was performed in five databases (Virtual Health Library, Health Systems Evidence, Epistemonikos, Medline/Pubmed, and the Cochrane Library) up to 2010. Studies published in Portuguese, Spanish, and English were included, to evaluate the effect of diets on the treatment of DM in adults. As a result, 31 systematic reviews were included, assessing: diets with modifications in carbohydrates; vegetarian or vegan diets; Mediterranean diets; fasting or intermittent fasting programs; diets with protein modifications; specific food plans; energy-restricted diets; and fat-modified diets. Among the included studies, the effects of different nutritional strategies on the outcomes of interest regarding DM were evaluated (glucose and glycated hemoglobin, and anthropometric measurements). The detailed methodology of this systematic review was published at: https://www.veredas.org/en/publications/#647, accessed on 15 September 2022.

### 2.5. Development of Dietary Guidelines Recommendations for Adults with DM

Dietary guidelines’ recommendations specifically for adults with DM, with a flowchart to assist the PHC professionals in the application of the dietary guideline, were elaborated based on the final list of extracted recommendations in step 3 and in accordance with the systematized literature search in step 4. 

### 2.6. Validation of the Protocol

**Figure 1:**
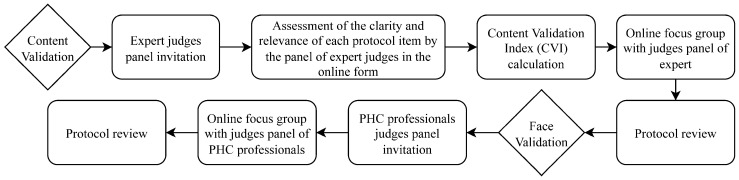
Flowchart of the content and face validation steps. PHC: Primary Health Care.

After developing the first version of the protocol according to the previous steps, the instrument went through validation, consisting of content and face validation. The validation steps are summarized in Figure 1.

#### 2.6.1. Content Validation

A panel of expert judges in DM care, food guidelines, and nutritional care in PHC was formed by snowball sampling [10]. The judges received the methodological guidance and the evidence synthesis (the result of step 4) by email to support the validation process and the online form to assess each protocol item. They evaluated the items considering their clarity and relevance, according to the 4-point Likert scale (1-the item is unclear/relevant; 2-major revisions are needed to make the item clear/relevant; 3-minor revisions are necessary to make the item clear/relevant; 4-the item is clear/relevant). If any item received less than 3 points, a written justification or suggestion for revision was required. 

After answering the online form, the judges were invited to an online focus group to assess their overall impressions of the protocol conducted by guiding questions such as: 1. What is your opinion about the proposal to draft a Protocol with this scope? 2. Are the language and content adequate? 3. Do you think the recommendations are based on the best available scientific evidence on diet and DM or do you have any additional suggestions? 4. Do you believe that the protocol is coherent with its objectives, considering whom it is intended for (health professionals) and where it should be used? 5. Do you have any additional improvement suggestions? All contributions were analyzed and changes in the protocol then led to the new version.

After content validation, the suggestions were categorized for analysis according to their topic [11]. Then, the research team revised the protocol, including or excluding the suggestions [7]. All modifications made to the protocol were appropriately justified. Then, a new version was carried out proceeding with the face validation [12].

#### 2.6.2. Face Validation

To validate the protocol for its understanding of the content and the applicability to potential users, graduated health professionals who work in PHC in all five macro-regions of Brazil (North, South, Midwest, Southeast, Northeast) were invited to debate online in separated focus groups. The face validation focus group was taken according to guiding questions such as: 1. Thinking about your practice, what are the main difficulties and challenges that people with DM report related to food? 2. Did you already know the DGBP? What did you think of the proposal to prepare a protocol related to the Guide? 3. Can the protocol help with dietary guidance in your professional practice? 4. Considering the reality in PHC, do you think that the protocol recommendations addressed the needs of people with DM who attended PHC? 5. Regarding the protocol’s approach to social stigma and the reflections on the “blaming” of the individual with DM, do you consider them to be relevant? 6. Does the protocol address specific characteristics of your region of the country? 7. In addition to what was said, would you like to make any other comments, suggestions, or limitations?

After face validation conclusion, all suggestions were evaluated by the research team, and the final version of the protocol was carried out. 

The research team mediated both content and face validation focus groups and recorded and transcribed upon permission of the participants. 

### 2.7. Data Analysis

The expert judge agreement was assessed by a Content Validity Index (CVI) for each protocol item obtained in the completed online form. CVIs measure the degree of agreement of the experts regarding the clarity and relevance of each protocol item. The CVI was calculated by the proportion of items received in grades 3 and 4, divided by the total number of specialists, for clarity and relevance separately. The total Content Validity Index (tCVI) was also assessed by the average of all CVIs. It was considered an appropriate item if CVI and tCVI > 0.80 [13]. 

### 2.8. Ethics Aspects

The study was approved by the Ethics Committee for Research with Human Beings of the Faculty of Health Sciences, University of Brasília. All participants signed the Free and Informed Consent Form. 

## 3. Results

### 3.1. Development of the Protocol 

The protocol format consisted of the following sections: 1. Introduction—with a brief elucidation of the clinical and epidemiological aspects of DM, the DGBP, and the protocol’s aim; 2. “How to use the protocol?”—step-by-step instructions on how to use the protocol for Brazilian PHC professionals; 3. The instrument for assessing Food Consumption—The Food Consumption Markers instrument chosen was the form used in the Brazilian Food and Nutrition Surveillance System (in Portuguese: Sistema de Vigilância Alimentar e Nutricional (SISVAN)) [14], considering the ease in fulfilling it and already used in PHC instruction [15]; 4. Flowchart—A circle model flowchart centered on the patient’s decisions alongside the PHC professional was elaborated to guide the health professional to dietary recommendations, reinforcing the patient’s autonomy in their health care; 5. Dietary recommendations; 6. Final messages.

The recommendations extracted from the DGBP were centered on the food groups according to the NOVA classification [5], enhancing unprocessed and minimally processed foods as the basis of the diet, limiting the consumption of processed food, and avoiding ultra-processed food; as well as eating regularly and carefully, in appropriate environments, and whenever possible with company; to shop for unprocessed food or, when away from home, to eat in places with a variety of in nature and minimally processed food; encouragement to develop and exercise culinary skills.

Because the particularities of dietary care in DM were not covered by DGBP, the most relevant recommendations were selected from the Diabetes Brazilian Society guidelines [8]. The following recommendations were considered: 1. Stimulate Mediterranean and Dietary Approaches to Stop Hypertension (DASH) dietary patterns; 2. Enhance the overall quality of food rather than the restriction of some group of nutrients; 3. Limit the consumption of rich foods in saturated fat and added salt/sugar; 4. Give preference to grilled, roasted, steamed, or raw preparations; 5. Recommend not to ban sucrose and foods containing sucrose, but to avoid consumption of hidden sugars in processed foods; 6. Fad diets such as low-carb diets are not recommended because the impact of this type of diet is still inconclusive; 7. Encourage dietary fiber intake (fruits, vegetables, legumes, cereals, and whole grains) [8].

Considering the literature review search and the recommendation from the DGBP and Diabetes Brazilian Society guidelines, the protocol reinforces the eating patterns based on low-fat content, low meat intake, with vegetables, fruits, whole grains, beans, rich in fiber and antioxidants, with low energy density and low glycemic index. Mediterranean and vegetarian eating-pattern diets show improvements in glycemic control and anthropometric measurements in adults with DM.

The literature review carried out regarding dietary guidelines evaluated low carb or very low carb diets (n = 18). Despite the weight loss promoted by diets, the effects on glycemic control were conflicting, and fewer studies investigated their adhesion or controlled potential confounding factors (e.g., medication). The detailed results of this review are published at: https://www.veredas.org/en/publications/#647, accessed on 15 September 2022.

Overall, the protocol included six main recommendations and final messages:

1. Recommendation 1—Encourage the daily consumption of beans.
2. Recommendation 2—Advise the avoidance of sugar-sweetened beverages.
3. Recommendation 3—Advise the avoidance of ultra-processed foods.
4. Recommendation 4—Advise the daily consumption of vegetables.
5. Recommendation 5—Encourage the daily consumption of fruits.
6. Recommendation 6—Encourage the person to eat in appropriate environments and with attention.

Final messages: additional guidance with specific guidance about fiber, meat, egg, dairy consumption, alcohol intake, fad diets, and management of hypoglycemic episodes; and a valuing of the eating practice already existing.

The recommendations contained the direct guideline, suggestions for variation, reasons, and possible obstacles, to increase the person’s adherence to the recommendation.

### 3.2. Content Validation

The panel of experts was composed of 19 participants, all female, with complete higher education in nutrition (5), medicine (3), public health (7), pharmacology (1), physiotherapy (1), and nursing (2).

Experts evaluated 28 protocol items through an online questionnaire between January and February 2022. Each expert judge evaluated all protocol items at once. Of the 28 items, 24 received total agreement (CVI = 1.0) for clarity, and 23 for relevance. Overall, all protocol items were adequate according to the average CVI cut-off point (>0.80) (Table 1). According to the evaluation of the CVI, the items that most needed changes were: “Recommendation 1: Consumption of beans-guideline”; “Recommendation 3: Ultra-processed food-Suggestion for variations”; “Recommendation 4: Legumes and vegetables–suggestion for variation”; “Final messages–additional guidelines”.

The main topics that emerged in the focus group and a summary of the reformulations carried out are presented in Table 2.

### 3.3. Face Validation

The face validation was composed of 12 PHC professionals, nurses from the Southeast (3), Midwest (3), and the North (1), physicians from the Southeast (1) and Midwest (1), a dentist from the Southeast, a psychologist from the Northeast, and a physiotherapist from the South.

In the focus group, PHC professionals suggested including in the “How to use the protocol?” section the identification of individuals who are in a vulnerable situation with difficulties in acquiring not only food but also adequate and healthy foods, as recommended in the DGBP (unprocessed or minimally processed). In the section “Recommendation 6—Appropriate environments?” suggestions were made to include the professional’s knowledge surrounding the territory, such as the identification of community gardens to indicate to health service users or verifying the possibility of crafting a garden with the users, aims to improve the consumption of unprocessed foods; and complementing the justification of the impacts and harms of restrictive diets, such as the potential for increased risk of binge eating, weight regain, and others. In the section “Recommendation 3—Ultra-processed food” they suggested including an expansion of examples of typical culinary preparations as a substitute for ultra-processed foods.

After the face validation, all suggestions previously described were contemplated (Table 3).

**Figure 2:**
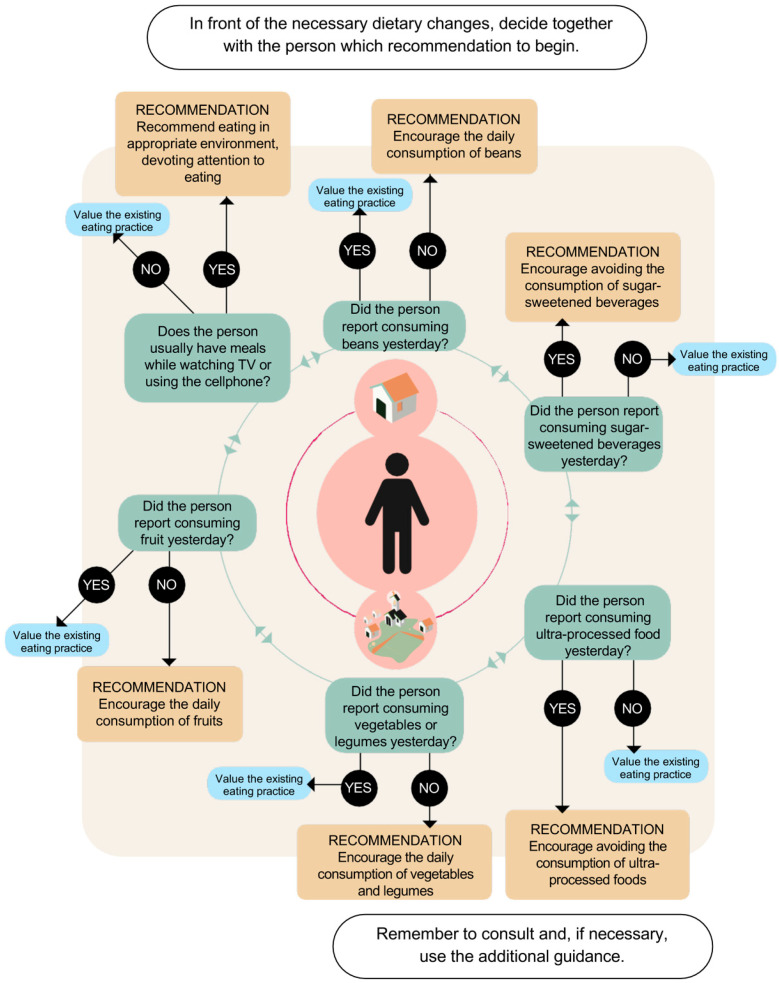
Directional flowchart of conduct for dietary guidelines for adults with diabetes mellitus, based on Food Consumption Markers.

The final version of the flowchart, after the considerations of PHC professionals, is presented in Figure 2. Overall, the professionals evaluated the protocol as useful and applicable in individual appointments with adults with DM assisted in Brazilian PHC. Hence, the final version of the protocol for the DGBP for adults with DM was concluded [16]. The published version of the protocol can be found on the Brazilian Ministry of Health website (in Portuguese): http://aps.saude.gov.br/biblioteca/index/MQ==/Mw== accessed on 15 September 2022.

## 4. Discussion

This is the first study to develop and validate a dietary protocol based on the DGBP for non-nutrition professionals working in PHC, presenting nutritional guidelines for adults with DM. Recent studies conducted by Jesus et al. [17] and Louzada et al. [7] developed dietary protocols also based on the DGBP for PHC professionals with a similar methodological design; however, they focused on dietary guidelines for the elderly and adults without comorbidities, respectively.

The organization of PHC based on the installation of Basic Health Units and multi-professional teams in delimited territories and with an enrolled population increases the decentralization and capillarity of the service of the Unified Health System, making it the preferred gateway to the health care system [18].

The dietary protocol developed contributes to disseminating dietary guidelines for people with DM to other non-nutrition professionals. Food and nutrition are determinants of the health–disease process, which can cross the practice of all health professionals. Even though nutritionists have specialized technical training in food and nutrition, all PHC professionals must be able to offer food-related care. Therefore, shared responsibility is proposed, from the perspective of interdisciplinary care [19]. It is important to reinforce that the protocol does not replace individualized dietary counseling by a nutritionist.

The development and elaboration of the dietary protocol provide support to assist PHC professionals to guide adequate and healthy eating for adults with DM. Studies indicate that when PHC teams prioritize actions to promote adequate and healthy food according to the national guidelines or strategies, the benefits to the health and nutrition of users are improved [20]. However, ensuring the implementation of this protocol in all Brazilian regions can be a challenge given the vast territory and the full schedule of PHC professionals. Therefore, it is still necessary for governmental efforts to train non-nutrition professionals and to disseminate the protocol for its effective implementation in clinical practice and consequently, the management of this chronic disease [21].

DM represents a significant economic burden on health systems, patients, and their families. In 2021, the total DM expenditure was estimated to represent 11.5% of total global health-spending [2]. On the country level, in 2021, Brazil was considered the third highest country with diabetes-related health expenditure (USD 42.9 billion), and the United States of America (USA), and China were the first and second countries, respectively, in the ranking [2].

To reduce the economic burden and the impact on the individual, family, and social sphere of the person with DM, dietary guidance is essential to manage and reduce the risk for diseases associated with diabetes, since food intake is one of the pillars of DM care [8]. The Brazilian Society of Diabetes (SBD) and the Brazilian Ministry of Health have already published specific materials for the guidance of people with diabetes, the carbohydrate counting manual, [22] and a guide for people with DM in PHC care, respectively [4]. Despite clarifying the clinical conduct of other PHC professionals, those materials do not include guidelines on healthy eating patterns, nor the food processing level (NOVA classification) and its consequences for health [4,22].

The dietary recommendation for promoting adequate and healthy eating present in the DGBP is directed at all Brazilian individuals, regardless of the presence of diseases. Recently, the recommendations presented in the DGBP based on NOVA classification have been associated with various health outcomes, [23,24] including diabetes-related ones [25,26]. The recommendation of avoiding ultra-processed and preferring unprocessed and minimally processed food has been associated with a reduced risk for type 2 DM [25,26]. In addition, this contributes to improving glycemic control by reducing the glycosylated hemoglobin in people with this disease [27], reinforcing the importance of the adaptation of these dietary guidelines for an adequate and healthy diet for the population with DM in PHC.

In addition to dietary guidelines, the protocol involves other important aspects to be considered in health care for people with DM, such as the stigma related to the disease and the language used in caring for people with DM. Stigmatizing experiences also occur in health care settings and can cause negative impacts on self-care (e.g., distress, depression, anxiety, and lower self-esteem) and willingness to seek health care support [28,29]. To prevent the stigma related to DM, professionals should reflect on their personal beliefs, language, and attitudes related to the disease throughout the care and counseling of their patients [30,31]. Therefore, the protocol stimulates the health professional to identify the stigma related to DM that may unconsciously manifest itself when carrying out dietary guidelines, for example, through judgments regarding food choices, and to rethink how to attend.

Another aspect of DM care that is contemplated in the present dietary protocol is the communication focused on the autonomy of individuals and recognizing the importance of collaborative work between the health professional and the person, rather than authoritarian attitudes. The way the health care professional communicates with people during the individual appointment plays a central role in making them feel respected and establishing a relationship of trust, which consequently favors engagement in their self-care and greater well-being [31,32,33].

This is the first study to develop and validate a dietary protocol based on Dietary Guidelines for the Brazilian Population for PHC non-nutritionist professionals in counseling adults with DM. The protocol is also the first to present instruments to assist individuals with DM to implement the recommendations in their daily lives, thus increasing the user’s adherence to the dietary guidelines. Moreover, the presented protocol was developed using meticulous and reproducible methods, and it was validated with the contribution of professional experts and potential users. The low adherence of PHC professionals to the face validation can be explained by the difficulty in reconciling their work schedules, and possibly by the low familiarity with the virtual meeting platform used in the focus group during the COVID-19 pandemic. However, even with the reduced number of PHC professionals, it was possible to ensure the participation of professionals from all Brazilian macro-regions allowing a diverse discussion. Fortunately, in the content validation stage, there was high adherence of different specialists such as nutritionists, doctors, nurses, among others, which allowed the integration of diverse perspectives in the care of people with DM.

## 5. Conclusions

This protocol provides specific recommendations for implementing healthy eating habits for the population with DM assistance in Brazil PHC, thus preventing further diabetes-related complications. In addition, the protocol also supports PHC professionals in providing general dietary guidance to these people, based on up-to-date scientific evidence for adults with DM. Thus, the dissemination and implementation of this protocol throughout the Brazilian territory are essential for the best management and dietary guidance of adults with DM in PHC, as well as its dissemination to other countries, to support the development of further dietary guidance protocols specific for the DM public in other types of health care systems.

## Figures and Tables

**Table 1:** Evaluation of the content validity index by the protocol items to use the Dietary Guidelines for the Brazilian Population (DGBP) for adults with diabetes mellitus, Brazilian, 2022.

	Content Validity Index (CVI)		
Component	Clarity (Min-Max.)	Relevance (Min-Max.)	Average CVI
Introductory Text	1.00	0.94	0.97
How to use the protocol?	1.00	1.00	1.00
Flowchart	1.00	1.00	1.00
Recommendation 1—Consumption of beans			
Guideline	0.94	0.94	0.94
Suggestion for variations	1.00	1.00	1.00
Reason	1.00	1.00	1.00
Obstacles	1.00	1.00	1.00
Recommendation 2—Sugar-sweetened beverages			
Guideline	1.00	1.00	1.00
Suggestion for variations	1.00	1.00	1.00
Reason	1.00	1.00	1.00
Obstacles	1.00	1.00	1.00
Recommendation 3—Ultra-processed food			
Guideline	1.00	1.00	1.00
Suggestion for variations	0.94	0.94	0.94
Reason	1.00	1.00	1.00
Obstacles	1.00	1.00	1.00
Recommendation 4—Legumes and vegetables			
Guideline	1.00	1.00	1.00
Suggestion for variations	0.94	0.94	0.94
Reason	1.00	1.00	1.00
Obstacles and strategies	1.00	1.00	1.00
Recommendation 5—Fruits			
Guideline	1.00	1.00	1.00
Suggestion for variations	1.00	1.00	1.00
Reason	1.00	1.00	1.00
Obstacles and strategies	1.00	1.00	1.00
Recommendation 6—Appropriate environments			
Guideline	1.00	1.00	1.00
Reason	1.00	1.00	1.00
Strategies	1.00	1.00	1.00
Final messages			
Additional guidance	0.89	0.94	0.92
Valuing the practice	1.00	1.00	1.00
Total-Content Validation Index (tCVI)			0.99

**Table 2:** Summary of the discussions and adjustments after content validation of protocol to use the Dietary Guidelines for the Brazilian Population (DGBP) for adults with diabetes mellitus, Brazil, 2021.

Recommendations	Emerging Topics	Sub-Topic Supported the Reformulation
Introduction	Contextualization	-Emphasize the high prevalence of overweight and obesity in the Brazilian population with diabetes according to a recent national Brazilian survey (in Portuguese: Sistema de Vigilância de Fatores de Risco e Proteção para Doenças Crônicas por Inquérito Telefônico (VIGITEL)).
	PHC flow care	-Highlight the importance of the lines of care, which trace the individual care flow, and reinforce the Health Care Network available in the care of the person with diabetes.
	Diabetes Complications	-Include the risk reduction in DM complications, in addition to only glycemic management, as an advantage of the protocol implementation.
	Carbohydrate counting	-Inclusion request, considering the PHC reality in the orientation of persons who perform or have doubts about carbohydrate counting and its relationship with the use of insulin.
How to use the protocol?	Food consumption markers form	-Assistance in locating food consumption markers within the PHC Information System.
	People’s autonomy in their care process	-Valuing and strengthening adequate and healthy practices that a person with DM already has.
	Diabetes stigma	-Relevance of health care professionals in preventing DM stigma when treating people with DM.
	Equity	-Strengthening feasible guidelines and being attentive to cultural and socioeconomic issues.
	Flowchart	-Reinforcing that the process must be collaborative, between the professional and the person, from the decision of which dietary recommendation begins.
Sugar-sweetened beverages	Diet, light, and zero	-Insertion of examples, considering the relevance of strengthening that such foods are ultra-processed, and their consumption is not recommended.
	Consumption	-Encouraging the gradual substitution of chocolate-sugar beverages to cocoa powder.
	Foods	-Discouraging the consumption of sweeteners, due to their classification as ultra-processed food.
Ultra-processed food	Foods	-Expanding examples of ultra-processed food.
	Foods	-Expanding examples of different types of cereal according to their carbohydrate content and their association with other foods and culinary preparations.
	Consumption	-Reinforcing the non-stimulating of dessert consumption, but for those with this habit, recommended to substitute ultra-processed food for unprocessed or minimally processed options.
	Consumption	-Reinforcing the lower impact of consumption of sweet culinary preparations right after meals on blood glucose, rather than their isolated consumption. -Stimulating the avoidance or reduction in the consumption of ultra-processed sweet foods.
	Consumption	-Including vegetarian culinary preparations as substitutes for ultra-processed food, considering the different contexts and food preferences.
	Consumption	-Reinforcing the importance of analyzing food labels for adequate and healthy food choices.
	Foods	-Expanding examples of unprocessed food as substitutes for ultra-processed food.
Legumes and vegetables	Regional foods	-Expanding examples of regional foods.
	Consumption	-Reinforcing the consumption of foods that grow under the ground (roots) in the context of healthy eating.
	Consumption	-Demystifying the impossibility of consuming two foods from the root and tubercle group in the same meal, however, recommend reducing the portion of each of these foods.
	Food access	-Reinforcing avoiding eating at places with a predominance of ultra-processed food.
Fruits	Glycemia management	-Including examples of food rich in dietary fiber to combine with fruits for better glycemic control.
	Consumption	-Including practical strategies to stimulate fruit intake daily.
Appropriate environments	Equity	-Considering the different socioeconomic realities with a view to providing feasible guidelines.
Final messages	Fad Diets	-Inclusion of the impacts and damages of long periods of fasting on blood glucose.
	Low-carb Diets	-Reinforcing the low adherence to a low-carb diet in long-term periods and the low high-quality evidence of their safety.
	Glycemic management	-Reinforcing the strategy of associating different food groups in the same meal to promote a better glycemic control.
	Food ingredients	-Expanding the regional food examples.
	Hypoglycemia episodes	-Reinforcing the investigation of possible causes for the hypoglycemia episodes. -Reinforcement of the importance of maintaining medication with adequate dosages and meal planning. -Encouraging carbohydrate counting as a strategy to decrease the risk of hypoglycemia.
	Hypoglycemia episodes	-Insertion of guidelines for the management of hypoglycemic episodes.

Abbreviations, DM: diabetes mellitus; PHC: primary health care.

**Table 3:** Summary of the discussions and adjustments after face validation of protocol to use the Dietary Guidelines for the Brazilian Population (DGBP) for adults with diabetes mellitus, Brazil, 2021.

Recommendations	Emerging Topics	Sub-Topic That Supported the Reformulation
How to use the protocol	The autonomy of people with DM in their care process	-Reinforce valorization and encourage active participation throughout their care process.
	Food ingredients	-Encouraging and providing strategies for people with DM to access unprocessed and minimally processed foods, regardless of socioeconomic status.
Ultra-processed foods	Culinary preparations	-Expanding the examples of regional culinary preparations to replace ultra-processed foods. -Reinforcing the inclusion of protein-rich foods in culinary preparations, to improve glycemic control.
Final Messages	Fads diets	-Elucidation of the impact and potential harm of restrictive diets for people with DM.
	Food production and access	-Encouraging people with DM to make a vegetable garden in their homes or communities.

## Data Availability

The data presented in this study are available on request from the corresponding author.

## Abbreviations

PHC	Primary Health Care
DM	Diabetes Mellitus
DGBP	Dietary Guidelines for Brazilian Population
FHS	Family Health Strategy
SUS	Brazilian Unified Health System (In Portuguese: Sistema Único De Saúde)
CVI	Content Validity Index
VIGITEL	Surveillance System for Risk and Protection Factors for Chronic Diseases by Telephone Survey (In Portuguese: Sistema de Vigilância de Fatores de Risco e Proteção para Doenças Crônicas por Inquérito Telefônico)
